# Recent Advances in Physical Post-Harvest Treatments for Shelf-Life Extension of Cereal Crops

**DOI:** 10.3390/foods7040045

**Published:** 2018-03-22

**Authors:** Marcus Schmidt, Emanuele Zannini, Elke K. Arendt

**Affiliations:** 1 School of Food and Nutritional Sciences, University College Cork, Western Road, T12 Y337 Cork, Ireland; marcus.schmidt@ucc.ie (M.S.); e.zannini@ucc.ie (E.Z.); 2 Alimentary Pharmabotic Centre Microbiome Institute, University College Cork, T12 Y337 Cork, Ireland

**Keywords:** cereal grains, shelf life, spoilage microorganisms, mycotoxins, physical decontamination

## Abstract

As a result of the rapidly growing global population and limited agricultural area, sufficient supply of cereals for food and animal feed has become increasingly challenging. Consequently, it is essential to reduce pre- and post-harvest crop losses. Extensive research, featuring several physical treatments, has been conducted to improve cereal post-harvest preservation, leading to increased food safety and sustainability. Various pests can lead to post-harvest losses and grain quality deterioration. Microbial spoilage due to filamentous fungi and bacteria is one of the main reasons for post-harvest crop losses and mycotoxins can induce additional consumer health hazards. In particular, physical treatments have gained popularity making chemical additives unnecessary. Therefore, this review focuses on recent advances in physical treatments with potential applications for microbial post-harvest decontamination of cereals. The treatments discussed in this article were evaluated for their ability to inhibit spoilage microorganisms and degrade mycotoxins without compromising the grain quality. All treatments evaluated in this review have the potential to inhibit grain spoilage microorganisms. However, each method has some drawbacks, making industrial application difficult. Even under optimal processing conditions, it is unlikely that cereals can be decontaminated of all naturally occurring spoilage organisms with a single treatment. Therefore, future research should aim for the development of a combination of treatments to harness their synergistic properties and avoid grain quality deterioration. For the degradation of mycotoxins the same conclusion can be drawn. In addition, future research must investigate the fate of degraded toxins, to assess the toxicity of their respective degradation products.

## 1. Introduction

At a time of rapid growth in global populations, sufficient nutritional supply to humanity has become increasingly challenging. On the basis of their long tradition as global staples of the human diet and livestock feed, agricultural crops such as cereals will have a key role in satisfying this growing nutritional need. However, global agricultural area is limited, making it difficult to expand cereal production. Considering that approximately 15% of all cereals worldwide are lost due to microbial pests [1], the most sensible approach to combat this issue is to increase both food safety and sustainability to reduce economic losses. Pre- and post-harvest microbial spoilage counts as one of the predominant factors in crop loss all over the world. Various strategies to prevent microbial contamination in the field have been investigated and reviewed by Oerke [2]. However, even the best management practices cannot completely eliminate the risk of contamination. Because of the permanent and ubiquitous presence of microorganisms and fungal spores in the environment, cereals always carry a certain microbial load when harvested. Additionally, climatic conditions, such as temperature and humidity, that are not under human control may be crucial for contamination with moulds [3]. Therefore, appropriate post-harvest crop treatment, before and during storage, is as important as pre-harvest strategies in the prevention of microbial spoilage. Thus, this review is focused exclusively on research regarding post-harvest treatments.

**Figure 1:**
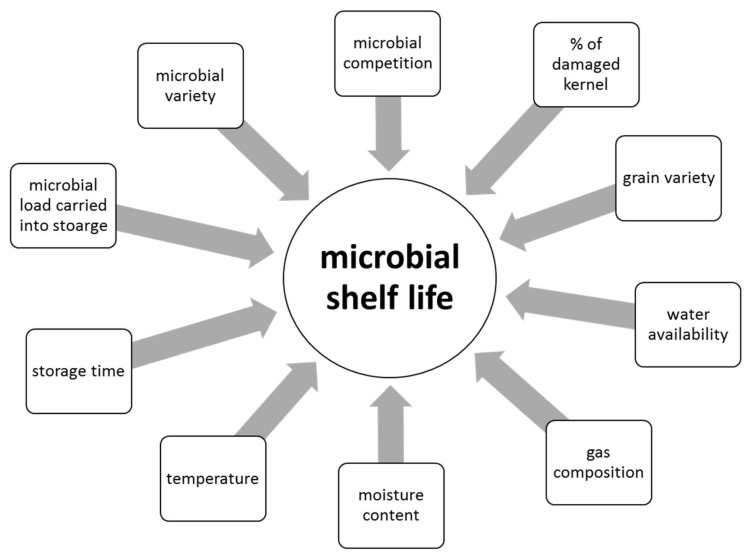
Biotic and abiotic factors influencing the microbial shelf-life of cereal grains during storage.

Depending on climatic conditions during growth, grains carry a microbial load with a high diversity of potential spoilage organisms when harvested. In addition, post-harvest contamination during transport is possible. This microbial load consists of bacteria, yeasts, and filamentous fungi belonging to many different genera. The activity of these micro-organisms during storage and, accordingly, the shelf life of the crop is dependent on a range of factors, as illustrated in Figure 1. Amongst the most influential parameters are moisture content and water availability during storage. As a result, grains are usually stored at low moisture contents of 12–13% and a water activity of <0.70 [4]. However, cereals are usually traded as wet weight and thus inefficient drying systems can lead to microbial spoilage during storage. Furthermore, even if dried properly, some xenerophilic species of *Aspergillus* can still develop during storage, resulting in quality deterioration and mycotoxin accumulation [5].

In addition, conventional storage systems, such as silos, are often cost intensive and inflexible as to volume. In these rigid systems, due to the inappropriate size for the amount of grains stored, the environmental conditions in the headspace cannot be controlled. Thus, it is likely that suitable conditions arise which promote microbial growth and the production of toxic metabolites [6]. As shown in a recent study conducted by Schmidt et al. [7], if the conditions during storage are suitable, a minimal fungal field contamination can rapidly evolve into a serious consumer health hazard. 

The biggest microbial threat during storage is displayed by filamentous fungi, mostly belonging to the genera *Aspergillus* and *Penicillium*. This is largely a result of their relative tolerance to low water activities and the production of mycotoxins, which are secondary fungal metabolites with toxic effects on humans and animals. In addition, these fungi induce a loss of nutritional value in grain, produce off-odours, and result in reduced baking and milling quality [4]. Although *Fusarium* spp. are typical field pathogens and unlikely to develop during storage, previously produced and accumulated mycotoxins remain a serious issue during cereal storage and processing [8]. Hence, in addition to the living organisms, a broad variety of mycotoxins must be countered during post-harvest treatments, as the prevention of their production is not always possible. Table 1 shows the most commonly reported mycotoxins of cereal crops and the producing fungal genera. It has been reported that approximately 25% of the global cereal crops, equivalent to over 500 million tons per annum, are contaminated with mycotoxins and thus present a potential consumer health hazard [9]. In contrast to fungal mycelia, mycotoxin-contaminated grains often do not vary visually from clean grains and are therefore difficult to identify and eliminate.

Previous studies have established that mycotoxins are primarily located in the husk layers of grains. A study conducted by Vidal et al. [10] showed that wheat bran obtained from a Spanish market contained substantial amounts of various mycotoxins, mainly deoxynivalenol (DON) and zearalenone (ZEA). The authors concluded that the production of whole grain products from these wheat samples would constitute a substantial consumer health hazard. However, since wheat bran is rich in dietary fibre, there is a high interest in exploiting its application in food and feed products because of its potential health-promoting properties. In addition to these *Fusarium* toxins, different toxins produced by storage fungi, namely aflatoxins (AFs), ochratoxin A (OTA), and citrinin are commonly found as contaminants in cereals (Table 1). Finally, bacterial spoilage organisms must also be considered to ensure sufficient grain shelf life. Although most bacteria are unlikely to grow under conditions commonly applied to grain storage, their presence can result in significant quality deterioration during subsequent processing or in the final product [11].

In recent years, the consumer desire for more natural, less processed foods with fewer or no chemical additives has increased enormously; however, the requirement for the maintenance of the highest safety standards remains. This increases the emphasis on physical and microbiological treatments to control post-harvest microbial spoilage in cereals [12]. In addition, the application of both physical and microbiological decontamination methods into necessary food processing procedures while simultaneously enabling a “clean label” has attracted increasing interest from both the industry and researchers. Various approaches for bio-preservation, particularly the use of antifungal LAB (lactic acid bacteria), have been investigated and reviewed elsewhere [5,13,14]. In contrast, this article focuses on the recent advances in novel physical decontamination methods.

Physical grain treatments, including dry and wet heat, ionizing and non-ionizing irradiation, high hydrostatic pressure and modified atmosphere packaging, were critically reviewed as to their suitability to eliminate viable forms and spores of both food spoilage bacteria and fungi commonly found on cereals. Furthermore, the treatments’ potential to remove previously produced mycotoxins as well as the impact on grain quality, viability and technological performance were evaluated based on the existing literature. This allowed for the identification of future research needs as well as possibilities for industrial application of the treatments discussed. It should be mentioned that classical physical treatments, such as milling, sorting, and hulling are not covered in this review, as these methods are well established and industrially applied. Therefore, they largely have not been the subject of recent research.

## 2. Modified Atmosphere Packaging (MAP)

Modified atmospheres have been investigated for the storage of cereals intended for food and feed. While fungi responsible for grain deterioration during storage often are considered obligate aerobes, many are microaerophilic. Hence, they can develop in niches where other species cannot grow and therefore can dominate in grain ecosystems. In many cases, the oxygen level must be <0.14% and the carbon dioxide level >50% to achieve significant growth inhibition [15,16]. Certain species, such as *P. roqueforti* Thom and some *Aspergillus* spp., can grow and infect grains even at >80% carbon dioxide, if at least 4% oxygen is present. In addition, post-harvest systems also use (oxygen-free) nitrogen to prevent grain deterioration [17]. However, it must be noted that results obtained in different sample systems are very difficult to compare, as the tolerance to low oxygen and high carbon dioxide levels is highly dependent on the sample matrix and water availability. Accordingly, low water availability has been reported to increase the sensitivity of microorganisms to the modified atmosphere. While MAP is used to control microbial spoilage and insect pests in moist grains during storage, different threats require different treatment conditions.

Exposure of various toxigenic fungi to ozone gas (60 µmol/mol) for up to 120 min resulted in significantly reduced growth and spore germination for *Fusarium graminearum*, *F. verticillioides* (Sacc.) Nirenberg, *Penicillium citrinum* Thom, C., *Aspergillus parasiticus* Speare, and *A. flavus* Link. However, the efficiency of the treatment was strongly dependent on the specific strain. *F. graminearum* was found to be particularly sensitive, as its growth was totally inhibited after exposure for 40 min, whereas *F. verticillioides* growth after 120 min of exposure was only slightly reduced [18].

However, very little research on modified atmosphere packaging has been conducted in recent years. This can be attributed to various reasons. Firstly, storage conditions under high carbon dioxide and low oxygen levels are difficult to apply to a conventional silo storage system. Additionally, environmental conditions, such as temperature, moisture content, and water availability determine the gas composition required to achieve sufficient microbial inhibition. The biggest potential drawback of this technology is that the microorganisms are not killed and therefore can induce product spoilage during subsequent processing. In addition, the removal of mycotoxins produced in field or the inactivation of microbial enzymes is not possible. In addition, microbial inhibition is substantially dependent on the fungal or bacterial strain. As cereals after harvest carry a wide range of microorganisms, it also appears very difficult to predict the success on a specific crop. Therefore, recent research to prevent post-harvest microbial spoilage has shifted towards novel and more flexible methods for application.

## 3. Thermal Treatments

Thermal treatments, including pasteurisation and sterilisation techniques, are the most regularly used treatment for the inactivation of microorganisms and enzymes in the food industry [19]. For post-harvest application of heat treatments various approaches, such as hot water dips, hot dry air, or superheated steam, are possible using different time-temperature combinations [20]. Table 2 summarises heat treatments reported to have been successful for microbial inactivation or toxin degradation and the side effects on the sample. 

### 3.1. Dry Heat Treatments

Described as an alternative to chemical grain disinfection, several applications of dry heat in the form of hot air treatments were studied for the ability to control fungal spoilage without compromising the kernels’ viability [25]. Contradictory results have been reported as to the effects of dry heat treatments on the cereal’s sensory quality and nutritional value. Several authors reported no impact on technological performance and enzymatic activities after nine days at up to 100 °C [21,22]. In contrast, Gilbert et al. [25] found a significant loss in seed viability after treatment for five days at 90 °C.

For inhibition of various fungal strains on wheat grains, time-temperature combinations of 15 day/60 °C, 5 day/70 °C and 2 day/80 °C, respectively, were found to inhibit fungal growth and spore germination completely without compromising the grain’s viability [25,30]. When treating barley at the same temperatures, the fungi were completely inhibited after 21 days, 9 days, and 5 days, respectively. In contrast to wheat, after nine days at 70 °C, the germination capacity of barley was substantially reduced.

Although bacterial spoilage organisms, such as *Bacillus* spp., are unlikely to cause grain quality deterioration during storage, they have to be inactivated prior to grain processing to avoid subsequent product spoilage [11]. However, no studies specifically investigating the heat inactivation of grain spoilage bacteria post-harvest are available to date. In general, bacteria are reported to be less heat stable than fungal mycelia and it can therefore be assumed that they are also inactivated with the abovementioned antifungal treatments [31].

Thus, dry heat treatment prior to grain storage shows potential for the prevention of post-harvest microbial spoilage based on the heat inactivation of the microbes and the reduction of the grain’s moisture content and water availability, which further supports microbial inhibition [32]. 

Depending on the processing conditions and sample matrix (i.e., the presence or absence of yeast), reports suggest a substantial reduction in the occurrence of mycotoxins during heating. However, cereal flours currently are not always heat treated during processing and this approach is very inconsistent. Therefore, an efficient pre-storage treatment to degrade mycotoxins is essential [33]. In general, conventional heat treatment is not suitable for detoxifying crops contaminated with mycotoxins. Commonly found mycotoxins, such as aflatoxins, trichothecenes, OTA, and others reportedly possess great heat stability (>300 °C) and thus cannot be degraded by dry heat without seriously damaging the treated cereal. To date, no studies reporting the total removal of mycotoxins by dry heat treatments in vitro or in situ are available. In addition, the reported partial degradation of different toxins by dry heat was always found to compromise the grain quality and viability. This indicates that dry heat can serve as a sufficient tool for the degradation of mycotoxins only in combination with other treatments [34]. In addition, the thermal degradation of mycotoxin into intermediate constituent products of unknown identity and toxicity must also be considered [35,36].

Available literature suggests that, depending on the target microorganism and cereal substrate, dry heat treatments have the potential to decrease the microbial load without compromising the grain quality. However, such treatments consume considerable energy and time, lasting up to several days. Furthermore, the conditions must be adjusted carefully to achieve satisfying results for both microbial decontamination and grain quality. In addition, the sole use of dry heat is unsuitable for killing spores of heat-resistant bacteria, as the applicable temperatures are too low and do not kill the grains. Hence, bacterial spores enter a dormant state and can germinate once suitable conditions return, particularly during cereal processing [26]. In terms of mycotoxin degradation, dry heat cannot serve as a sole treatment to efficiently remove mycotoxins produced in-field. The temperatures required are not feasible to maintain the grain’s technological performance; decomposition is always incomplete and the resulting degradation products could display a health risk of yet unknown potential. Finally, the efficiency of hot air treatments is further compromised by the particle size of the treated sample. Thus, it is less efficient for the treatment of whole grains than for treating the milled product.

### 3.2. Wet Heat Treatments

Compared to conventional hot air treatments, the use of hot steam appears to be a more efficient approach in terms of both time and energy for microbial decontamination and mycotoxin degradation (Table 2). In addition, food spoilage bacteria and fungi were found to be less heat resistant when in conditions with high water availability [37]. Apart from the classic saturated water steam (up to 100 °C), superheated steam (SS, up to 250 °C) has recently gained considerable interest as a rapid, non-destructive, and safe decontamination method [38]. Hence, recent advances and novel applications of SS will be the focus of this section. SS, having higher enthalpy than saturated steam, can quickly transfer heat to the material being processed, resulting in rapid temperature increase in the sample. In addition, SS is reported to contribute to better product quality. The major advantages of using SS for food processing include better product quality (colour, shrinkage, and rehydration characteristics), reduced oxidation losses, and higher energy efficiency [39].

Researchers using SS achieved microbial decontamination from vegetative forms of food spoilage fungi (*Aspergillus* spp., *Penicillium* spp., *Fusarium* spp.) and bacteria, such as *Escherichia coli* O157:H7, *Salmonella Typhimurium*, *Salmonella enteritidis* phage type 30, and *Listeria monocytogenes*, on different cereals. Treatment times of less than 60 s with water steam temperatures of 170 °C–200 °C were reported to be sufficient to reduce the microbial load below the respective limit of detection [26,27,28]. However, none of those treatments resulted in a significant reduction of sensory or nutritional quality. In contrast, corn treated with conventional steam at 82 °C had to be exposed for 60 min in order to achieve a significant reduction of the microbial load by 4-log units [23]. No results regarding the impact of the treatment on the grain quality were reported. As a result, despite the ability of both approaches to decontaminate cereals, the use of superheated steam is a much more time efficient solution.

Compared to their vegetative forms, fungal and bacterial spore inhibition presents a much bigger challenge. As mentioned above, bacterial endospores can lay dormant and germinate when conditions are favourable during downstream processing. Nonetheless, SS was found to be efficient at permanently killing spores of *Geobacillus stearothermophilus*. However, exposure times of up to 20 min are required to kill spores. This is significantly longer than the inactivation of vegetative microbes. Thus, the biggest reduction in spore viability was detected during the first 5 min of treatment, independent from the processing temperature [29].

In addition, SS was also investigated for the removal of mycotoxins from different cereal matrices. Partial degradation of DON upon SS treatment was reported by Cenkowski et al. [29]. The authors found that increases in steam temperature and exposure time correlated with higher degradation rates for DON. Thus, the highest treatment temperature (185 °C), combined with the longest treatment time (6 min), resulted in the highest reduction of DON, by 50%, which was found to be independent of the steam velocity. Furthermore, the reduction was found to be exclusively due to thermal degradation, rather than solubilisation and water extraction with the steam. However, no investigation of the treatment’s impact on the grain’s technological performance were undertaken in the study of Cenkowski et al. [29]. Likewise, the dry decomposition temperature of aflatoxins (approximately 270 °C) is known to be significantly reduced under moist conditions [40]. Consequently, SS treatments present a much more efficient and promising approach for AF (aflatoxins) degradation than conventional dry heating.

In conclusion, the application of wet heat and superheated steam were found to be very effective in the decontamination of cereals without compromising the grain’s technological quality and performance. However, despite extensive research, future work is still required to optimise the processing parameters which is dependent on the matrix present. The technological impact of treatments long enough to kill spores and degrade mycotoxins requires further investigation. Additionally, the fate of mycotoxins thermally degraded by the SS remains unclear. Thus, the degree of actual detoxification is still in question. Similarly, the possibility of degrading other toxins, such as patulin or bacterial toxins, requires closer investigation.

Finally, it is noteworthy that ultra-superheated steam (USS) has recently attracted considerable research interest. This technique employs temperatures of 400–500 °C. Exposure of different cereals, namely wheat, barley, and rye for 15 s to USS (actual contact temperature 210–250 °C) resulted in total inhibition of spoilage fungi and grain shelf-life extension without notable quality deterioration [24]. However, few studies have investigated the application of USS for the microbial decontamination of food commodities. Thus, the optimal conditions of use and the full potential of this method remain unclear. Further research is needed to clarify the suitability of USS treatments to decontaminate and potentially detoxify cereal grains post-harvest.

## 4. Ionizing Irradiation

Ionizing irradiation approaches largely are based on short waves of electromagnetic energy that travel at the speed of light. All treatments discussed in this section share the basic properties of electromagnetic radiation. Ionizing radiation treatment using either gamma rays or an electron beam (e-beam) is well established as a rapid, efficient, safe, and environmentally friendly technique for the reduction of food-borne diseases by destroying pathogenic and toxigenic microorganisms [41]. Their biggest advantage is their great penetrating power (inversely related to the frequency [42]), their high efficiency against various food spoilage organisms, and the absence of a rise in temperature in the treated sample [1]. In addition, irradiation treatments for food processing purposes are unconditionally regarded as safe for dosages of up to 10 kGy (1 Gy = 1 Joule of irradiation energy/kg sample matter, with 1 Joule = 1 $\frac{\mathrm{kg}\cdot m^{2}}{s^{2}}$) [43]. Due to the unit of irradiation dosage containing the ratio of energy per time and sample matter, treatments are commonly compared by means of dose and form of application only. Successful treatments with ionizing irradiation against various food spoilage organisms and toxins are summarised in Table 3.

### 4.1. Gamma Irradiation

This section discusses the application of irradiation to decontaminate cereals using gamma rays. These electromagnetic waves are usually produced by radioactive cobalt isotopes (^60^Co). The use of gamma rays for irradiation treatments is characterised by their high penetration energy and short treatment times.

For millet grains exposed to gamma radiation, no significant decrease in fungal incidence or spore germination was reported for radiation doses up to 0.5 kGy. However, at doses of 0.75 kGy or higher, the rate of fungal incidences and spore germination sharply decreased by over 80% and 2-log units, respectively [44]. Salem et al. [48] applied gamma irradiation ranging from 0.5–3.5 kGy to wheat grains prior to storage. A dosage of 1.5 kGy was found to be sufficient for at least a 90% reduction of *Aspergillus* spp., *Alternaria* spp., *Fusarium* spp., *Curvularia* spp., and *Helminthosporium* spp. immediately after the treatment. However, after six months of subsequent storage, the degree of inhibition (compared to the untreated control) was significantly reduced for all species apart from *Fusarium* spp. The authors also reported that higher irradiation doses resulted in higher inhibition rates. In particular, 3.5 kGy, resulting in total inhibition directly after the treatment, also showed total inhibition after six months against all fungi apart from *Aspergillus* spp. (96.4%). In contrast, Aziz et al. [49] reported higher irradiation doses, 4 kGy for barley and 6 kGy for wheat and maize, necessary for total inhibition of *Fusarium* spp. However, analysis of selected physical, chemical, and rheological properties of these grains prior to and after storage showed that, from a technological performance point of view, irradiation dosages higher than 1.5–2.5 kGy were not feasible. This also correlates with findings reported by other researchers [58,59].

Available literature suggests that the radio sensitivity of different fungal species appears to differ significantly depending on the reference consulted. These discrepancies likely result from countless influencing factors which require further research. These factors include the form of fungal contamination (mycelium or spores) and the moisture contents of spores or commodities. Although moist conditions promote fungal growth and spore germination, dry spores are considered more irradiation resistant. Furthermore, the age of spores and the nature of the matrix irradiated can have a significant influence on the radio sensitivity. In addition, the fungal strain and the temperature before, during, and after irradiation influence the treatment’s efficiency for each sample.

However, the most recent research on the application of gamma irradiation on cereals has focused on the degradation of mycotoxins in food and feed commodities. Several studies have investigated the degradation of aflatoxins (AFs) and ochratoxin A (OTA) in particular. Deberghes et al. [60] previously reported on the degradation of OTA in an aqueous solution due to gamma irradiation with 2–5 kGy. However, in situ degradation of mycotoxins such as OTA was found to be much more difficult. After gamma irradiation of wheat and sesame seeds using 15 kGy, degradation of OTA, AFB_1_ (Aflatoxin B1), AFB_2_, AFG_1_ (Aflatoxin G1), and AFG_2_ with reduction rates of 23.9%, 18.2%, 11.0%, 21.1%, and 13.6%, respectively, were found [52,61]. However, in both studies the application of lower irradiation doses showed no substantial reduction of AFs and OTA. Several other authors also reported similar results on various cereal matrices intended for food or feed use [53,54,62,63,64]. However, it must be noted that none of these studies took the moisture content of the seeds into consideration, which appears to have a critical impact on the radio stability of the toxins. Therefore, the meaningfulness of the results is limited. In another study, soybeans, corn, and wheat, with respective moisture levels of 9, 13, and 17%, showed no substantial reduction in AFs or OTA after irradiation dosages of up to 20 kGy [56]. Therefore, the success of AF degradation due to gamma rays is not dependent solely on the dose; the moisture content of the sample has a major impact on AF degradation. It is proposed that this is primarily due to the radiolysis of water, producing highly active radicals which can degrade AFs to compounds with lower biological activity [40,65]. Similar results were reported for the degradation of OTA in vitro and in situ. OTA showed great radiostability as a dry substance because irradiation with 8 kGy resulted in no noteworthy reduction. In contrast, when in an aqueous solution (50 ng/mL), significant reduction was achieved with just 2 kGy. Similarly, the degradation in moist wheat kernels (16% moisture) was substantially higher than in dry ones (11% moisture) when irradiated with 8 kGy [55].

On the basis of consulted literature, typical *Fusarium* toxins, such as DON, ZEN (Zearalenone), T-2 (Fusariotoxin T 2), and FB_1_ (Fumonisin B1) appear to be more radio sensitive than AFs and OTA. DON, ZEN, and T-2 toxin in soybeans, corn, and wheat were significantly reduced by irradiation with 10 kGy [56]. Irradiation of barley, wheat, and maize naturally contaminated with FB_1_ which resulted in a significant reduction (>85%) and total destruction of the toxin after exposure to 5 kGy and 7 kGy, respectively [49].

Nevertheless, irradiation results in degradation products of unknown identity and toxicity must be considered. Wang et al. [65] detected 20 radiolytic products of AFB_1_ after irradiation in a water/methanol mixture. To date, just seven of these products have been identified and six are considered less toxic than AFB_1_ due to the missing double bond on the terminal furan ring (Table 1). Given these results, it remains unclear if degradation of mycotoxins is responsible for the detoxification of the sample or if the resulting products are equally as toxic as the original substance. Furthermore, as the irradiation efficiency is highly dependent on water availability, the application in dry food matrices appears to be limited. Although AFs and OTA belong to the most commonly found toxins in cereal crops, the fate of other contaminants, such as DON, NIV (nivalenol), and ZEN requires further research.

In conclusion, gamma irradiation can reduce and potentially fully inhibit fungal and bacterial spoilage of grains during storage thus avoiding the production of mycotoxins. However, the irradiation dosages required for total inhibition of common storage fungi, such as *Aspergillus parasiticus*, are highly dependent on the sample matrix and fungal load. Necessary doses reported a range between 5 and 10 kGy. Unfortunately, such high irradiation dosages cause severe damage to the treated grains and are therefore not feasible from a technological point of view. On the other hand, a simple reduction of the microbial load is not sufficient either, as even minimal fungal contamination can lead to growth during storage. This ultimately results in huge economic losses and a potential consumer health hazard, as demonstrated by the authors in a previous study [7]. Therefore, gamma irradiation alone cannot serve as an efficient tool for cereal grain preservation during storage but could potentially be applied in combination with other treatments. However, for treatment of cereals purposed for animal feed, higher irradiation dosages are allowed. Thus, gamma irradiation may be a promising approach in the decontamination of animal feed.

### 4.2. Electron Beam Irradiation

The use of e-beam irradiation has several advantages over the use of conventional gamma irradiation. Table 4 summarises the comparison between gamma and e-beam irradiation. The main advantages include faster operation, lower irradiation dosages, and the use of electricity rather than radioactive materials to generate the electron, making the technology more flexible and easier to use. Unfortunately, its penetration power is lower, largely rendering it a tool for surface disinfection [1]. The general use of e-beam irradiation in the food industry, including the technological background and mode of action, was recently reviewed by Freita-Silva et al. [1]. Accordingly, this section will focus exclusively on recent advances in microbial decontamination using e-beam irradiation. With the exception of Europe, e-beam irradiation has been accepted and is widely applied towards the treatments of various food products worldwide, such as fruits and grains. Approximately 81,593 t are treated annually, out of which only 11 t are from Europe [66].

Microbial decontamination and mycotoxin degradation in vitro with the potential of application in situ has been previously reported [67]. The irradiation dose necessary for sufficient decontamination depends on the type and species of grains to be treated [68].

When dry split beans were subjected to e-beam irradiation (0, 2.5, 5, 10, 15 kGy) to control storage moulds, irradiation resulted in a dose-dependent decrease in fungal contaminants. High irradiation doses (10 and 15 kGy) resulted in a complete absence of fungi and undetectable levels of aflatoxins B_1_ and B_2_. In contrast, un-irradiated beans carried *Aspergillus niger* van Tieghem at the highest level (33–50%), followed by *A. flavus* (14–20%), and *Penicillium chrysogenum* Thom (7–13%). For total inhibition of fungal incidence, irradiation doses of 10 and 15 kGy were necessary. Irradiated split beans (10 kGy) also showed improved shelf life of up to six months without quality deterioration [50]. In contrast, on raw corn under comparable conditions, doses as low as 1.7, 2.5, and 4.8 kGy were found to be sufficient to fully inhibit the growth of *Penicillium* spp., *Fusarium* spp., and *Aspergillus* spp., respectively [41]. Researchers concluded that, with sufficient optimisation of the processing parameters, e-beam irradiation has considerable potential in the microbial decontamination of cereals. For the reduction of the natural microbial load in chestnuts, 6 kGy were found sufficient for decontamination while nutritionally valuable constituents remained unaffected [45,46,69]. Interestingly, e-beam irradiation of *Fusarium* spp.-infected barley with 6–10 kGy showed no significant reduction in fungal incidence and DON contents in the fresh barley. However, the resulting barley malt was reported to have significantly reduced DON content and fungal occurrence [51]. In another study conducted by Stepanik et al. [70], wheat grains and dried distillers grain were irradiated with up to 55 kGy, which resulted in a maximum DON reduction of 17%.

To date, no studies investigating the fate of mycotoxins exposed to e-beam irradiation are available. This is likely due to the lower energy value of this irradiation compared to gamma irradiation (Table 4). Thus, e-beam irradiation is unlikely to create enough energy for mycotoxin degradation. This applies in particular if the toxins are not exclusively located on the grain surface but also in the inner layers. Therefore, the application on ground almond flour with a dose as low as 1.5 kGy was found to be sufficient for total degradation of aflatoxins [57].

In conclusion, e-beam treatment shows potential to reduce the microbial load and content of mycotoxins produced in-field. However, depending on the sample matrix, microbial load, and target organism, the irradiation dose necessary is likely to exceed that generally permitted, i.e., 10 kGy [43], leading to legislative difficulties. Furthermore, the impact of various e-beam treatments on the grain’s sensory and nutritional properties have to be investigated further. Finally, difficulties such as the even treatment of the sample surface and the low penetration energy of the e-beam must be considered before any industrial application.

A variation of conventional e-beam irradiation is the use of so-called “soft electrons”. The term “soft” refers to the low energy of the electrons fired at the sample. This results in less impact on the sample in terms of sensory and nutritional value. However, this approach only can serve as a surface treatment, as the e-beam does not have sufficient energy to penetrate the deeper layers of the sample. The use of 3.3 kGy applied at soft electrons was reported to successfully eliminate *L. monocytogenes* from alfalfa sprouts [47]. However, due to the low penetration power, a small particle size and an even sample surface are crucial to ensure uniform treatment. Consequently, the irradiation treatment of soybeans was found to be more difficult, as 17 kGy was insufficient to eliminate the natural microbes present on the surface [71]. Thus, because of the difficult applicability, soft electrons show very little potential for industrial post-harvest decontamination of cereal crops but could have potential for flour treatment after milling.

## 5. Non-Ionizing Irradiation

This section reviews recent advances in non-ionizing irradiation treatments for microbial decontamination and detoxification with possible applications for cereals. Treatments that were successfully applied for the various target organisms or toxins are summarised in Table 5. Because of the non-ionizing character of the treatments discussed here, the impact on the grain quality is likely to be negligible. In addition, consumer acceptance for ionizing irradiation is relatively low due to misinformation [72]. Use of non-ionizing irradiation should increase consumer acceptance of the final products.

### 5.1. Ultraviolet (UV) Light

The antimicrobial properties of UV light are well investigated and, taking surface disinfection as an example, long established. However, the conventional application of UV light in the form of continuous exposure has numerous disadvantages. As the sanitizing effect is primarily attributed to the high ionizing energy of vacuum-UV (wavelength < 180 nm), the consumer acceptance is very low. The induced DNA damage, responsible for the microbial inhibition, also occurs in the sample which results in a substantial loss of quality. In addition, depending on the water content, substantial internal heating of the sample can occur. To avoid these unwanted side effects, recent research has focused on the application of pulsed UV light. In these studies, the treatment was carried out with numerous short flashes of light with a broad wavelength spectrum. Although the inhibitory effect was still attributed to the UV spectrum of light (wavelength 200–400 nm), microbial inhibition could be achieved with non-ionizing UV (>180 nm). At the same time, the undesired side effects were substantially reduced. Thus, pulsed UV light presents a novel, non-thermal, antimicrobial treatment with potential applications for food preservation. Therefore, this section is focused exclusively on the application of non-ionizing UV light. Microbial inactivation as a result of exposure to pulsed light, in vitro and in situ, has been reported by several studies and comprehensively reviewed by Oms-Oliu et al. [83]. 

Oms-Oliu et al. [83] demonstrated the successful inhibition of food spoilage fungi and bacteria with pulsed light when applied to various food matrices, including milk, honey, and fruits. However, only one available study investigated the application of pulsed light for pre-storage decontamination of cereals. Maftei et al. [76] reported up to a 4-log unit reduction for naturally occurring *Aspergillus* spp. in wheat grains. Treatment was carried out with 40 flashes of broad spectrum white light (180–1100 nm) with an overall energy release of 51.2 J/g. In addition, the authors reported that the same treatment with light of a narrower wavelength spectrum (305–1100 nm and 400–1100 nm, respectively) resulted in significantly less fungal inhibition.

Although pulsed UV light causes less damage to the sample than continuous UV irradiation, significantly reduced seed viability was reported nonetheless. Alongside the reduction of *Aspergillus* spp. from 10^5^ to 10 cfu/g, the seed viability was significantly reduced by 15% [76]. Consequently, despite showing the potential for microbial decontamination, further optimisation of the processing conditions is required to improve efficiency and make the treatment potentially suitable for industrial application.

However, inhibition of common food spoilage bacteria was generally found to be more difficult, as reductions of no more than 1-log unit could be achieved without substantial impact on the sample quality [83]. Furthermore, no studies investigating the inhibition of bacteria commonly found on cereals or in cereal matrices are available. However, Nicorescu et al. [79] achieved up to a 1-log unit reduction of *Bacillus subtilis* in a liquid medium and artificially contaminated spices. After the treatment, approximately 10^4^ cfu/g remained on the samples, despite an equivalent 90% reduction in the bacterial population. Thus, further research is needed to improve the antibacterial properties of pulsed UV light.

Several studies have also investigated the possibility of photodegradation of mycotoxins in vitro and in situ using UV and visible light. Treatment of different mycotoxins (OTA, OTB, and citrinin) in aqueous solution with light of various wavelengths (455, 470, 530, 590, and 627 nm) for five days resulted in significant degradation of all three toxins after exposure to the 455 nm and the 470 nm light [80]. In particular, light of wavelength 455 nm was found to be very efficient in terms of mycotoxin degradation. Subsequently, 455 nm light was applied in situ on artificially fungal-infected wheat kernels. After five days of exposure, the OTA and OTB levels were reduced by >90% compared to the untreated control [80].

In addition, the total aflatoxin content of various nuts could be substantially reduced by treatment with UV light (265 nm) for 15–45 min [81]. However, numerous factors were found to have a significant impact on the level of aflatoxin reduction. The most influential factors included the moisture content of the nuts, the toxin targeted, the exposure time, and the type of nut. The authors also reported higher resistance of AFB_1_ and AFG_1_ to the UV light compared to AFG_2_. For all toxins, increasing sensitivity towards the treatment could be attributed to increasing moisture levels and exposure time.

To evaluate the potential use of photodegradation for the detoxification of cereals, the fate of the degraded mycotoxins also must be considered. Liu et al. [84] proposed a pathway for the UV light-induced photodegradation of AFB_1_ after identification of the three main degradation products using UPLC-MS (ultra performance liquid chromatography-tandem mass spectrometer). Identification of the degradation products revealed that, from the two most important parts of the molecule in terms of toxicity, namely the terminal furan ring and the lactone ring (Table 1), only the latter was affected by photodegradation. In addition, the authors reported first order reaction kinetics and found the process to be independent of the initial toxin concentration (within 0.2–5 ppm) but directly related to the irradiation intensity. On the basis of these results, a subsequent study investigated the cytotoxicity of UV light degraded AFB_1_ in an aqueous solution [85]. The authors assessed the toxicity of the three previously identified degradation products using the Ames test. Interestingly, the cytotoxicity was reduced by 40% compared to native AFB_1_ but not fully eliminated. This leads to the question as to whether photodegradation can serve as a suitable tool for mycotoxin detoxification. Given that 60% of the initial cytotoxicity remained even after complete degradation of the toxin, alternative treatments are likely to be more suitable.

In conclusion, despite some potential for microbial decontamination and mycotoxin degradation, UV light appears to be difficult to apply without affecting sample quality. Although the use of pulsed light rather than continuous irradiation reduces the negative effects, no complete inhibition without quality loss has been reported. This applies in particular to the decontamination of food spoilage bacteria. For application on cereal grains, the uneven sample surface makes a reliable application even more challenging as a result of the shadow spots on the grains. In terms of mycotoxin degradation, it was shown that UV light was capable of total degradation of toxins such as AFB_1_, but the degradation products were found to remain cytotoxic. Therefore, it appears an unsuitable method for mycotoxin detoxification.

### 5.2. Microwave Treatments

Microwave technology is widely used in the food industry and offers several advantages, including safety, high efficiency, and environmental protection, but often affects food quality [74]. Microwaves are defined as electromagnetic waves with frequencies ranging from 300 MHz to 300 GHz. The mechanism of microbial inhibition is primarily based on the internal heating of the sample resulting from molecular movement in the pulsing electromagnetic field. This leads to the denaturation of proteins, enzymes, and nucleic acids. This implies the risk of losing enzymatic activities in the grain, activities which are essential for downstream processing steps [74]. However, with optimised processing conditions, microwave treatment has the ability to fully inhibit microbial growth on cereal grains without compromising the grain’s germination quality [86]. Therefore, the impact of various factors, such as moisture content, microbial load, or sample matrix, need to be investigated further to determine the optimal processing conditions for each cereal matrix.

Available literature suggests a microwave treatment for up to 10 min with an energy output of 1.45 kW with 2450 MHz results in a very minor reduction of total AFs, including AFB_1_ [53]. However, the possibility of inhibiting the growth and spore germination of *Aspergillus* spp. in cereals and nuts by 3-log units, without significant quality deterioration, was reported by different authors [77,78]. A treatment time of 120 s with 2450 MHz and 1.25 kW was found to be sufficient. Consequently, due to the fungal inhibition, the amount of mycotoxins produced was also significantly lower.

Furthermore, non-thermal inactivation of microorganisms resulting from repeated exposure to sub-lethal doses of high frequency microwaves has been reported [74]. The lethal effect of low-dose microwave radiation (LDMR) on spoilage microorganisms was a result of a disruption of the cell membrane and induced DNA damage rather than protein denaturation. Thus, the mechanism by which LDMR causes fungal death is different from a conventional heating treatment [74]. However, in a study conducted on *A. parasiticus*, the severity of DNA injury was found to increase with rising temperatures. Thus, the inactivation effect is still partially related to the processing temperature. However, few available studies have investigated this topic. Thus, future research is needed to exploit this technology through the establishment of non-thermal, microwave-based, microbial hurdle processes that do not compromise the grain quality.

Overall, microwave treatment, because of internal heating, shows little potential for the microbial decontamination of cereals. This is primarily a result of heat-induced damage to the sample. The same conclusion must be drawn for the microwave-induced degradation of mycotoxins which possess high heat stability. However, the concept of non-thermal microbial inactivation appears much more promising. However, as research on this approach is in its infancy, it is not yet possible to predict the full potential and applicability to industrial grain decontamination process until more extensive research has been conducted.

### 5.3. Ultrasonication

The term ‘ultrasound’ (US) describes sonic waves with frequencies above the human audible range and are generally divided into two categories: high frequency ultrasound and power ultrasound. The former uses high frequencies of 2–20 MHz with low sound intensity (0.1–1 W/cm^2^) and is predominantly used in food quality analysis and medical imaging. In contrast, power ultrasound, or high-intensity ultrasound, is characterised by low frequencies (20–100 kHz) but high sound intensity (10–1000 W/cm^2^). Research on the inactivation of enzymes and microorganisms has predominantly focused on the application of high power ultrasound, considered a promising, novel, and non-thermal approach for microbial disinfection of various surfaces and food matrices [87]. In contrast, high frequency US shows much less potential for microbial decontamination. Therefore, this review focuses on power ultrasound exclusively.

Only a few studies have investigated the sole application of US e for microbial decontamination with contradictory results. Butz and Tauscher [88] demonstrated that US alone does not sufficiently inactivate food spoilage microorganisms, as the inactivating effects are not severe enough. In contrast, Chemat et al. [73] reported that, if the acoustic power applied is sufficiently high (>60 W/cm^2^), even US alone can induce cell rupture and thus microbial inactivation. Successful decontamination using US was only achieved under laboratory conditions and would be difficult to apply on an industrial scale. Most data available suggests that US alone is a very inefficient and energy consuming treatment for microbial disinfection and therefore must be used in combination with other treatments, such as sanitizing chemicals or heat [89]. Furthermore, the generation of such high-power US requires an immense energy input and is relatively inefficient compared to other techniques. This is further supported by O’Donnell et al. [19] who described the challenges encountered by the industrial scale-up of US technology. In addition, the application of this technology to cereal grains could prove difficult, as the treatment has to be carried out in a liquid medium. Therefore, it would be crucial that the cavitation of the liquid around the grains is evenly distributed.

In general, the biggest potential of US is in combination with mild heat or sanitizing agents, which have been shown to have a synergistic effect by several authors [90,91]. Herceg et al. [75] achieved total inhibition of *Aspergillus* and *Penicillium* spp., after exposure to ultrasound for at least 6 min at 60 °C when the applied power was ranging between 20 and 39 W/cm^2^. However, these results were achieved in liquid sample matrices in which the US waves can easily travel, resulting in an evenly distributed cavitation effect. But for possible application on cereal crops, it remains unclear if enough cavitation throughout the whole sample can be generated for a noteworthy disinfection effect. Thus, a small sample size appears necessary to provide uniform cavitation. Furthermore, the grains would be required to be in a “washing solution”, which produces a further challenge to the industrial use of this technology.

No available studies have investigated the application of US to decontaminate solid foods. Chemat et al. [73] recently reviewed the application of US in the food industry and its advances for decontamination of liquid food systems. However, chemical disinfection supported by US is likely to result in synergistic effects. Thus, it is apparently a promising improvement over conventional chemical disinfection (reduction of treatment time and unpleasant side effects) and therefore should be considered for further investigations. Ultrasound could be used to provide the energy necessary to form free radicals as reactive species and so support the disinfection efficiency of commonly used surface sanitizers. In particular, hydrogen peroxide- or sodium hypochlorite-based disinfection of food or food contact materials can be efficiently supported by US, creating more hydroxyl- and hypochlorite radicals, respectively. Furthermore, the use of US can substantially increase the speed and efficacy of conventional food preservation methods such as sterilisation and pasteurisation. This would allow a reduction in the processing time and temperature and therefore reduce undesirable side effects, such as changes in taste, colour, and nutritional value [73].

Few studies have investigated the impact of ultrasonication on mycotoxins. Lindner and Hasenhuti [82] reported the successful degradation of trichothecenes in contaminated corn while the technological performance of the treated samples remained unchanged. However, as discussed earlier, for the combination of gamma irradiation with chemical sanitizers, the production of hydroxyl radicals can lead to chemical degradation of aflatoxins. Theoretically it should be possible to produce such radicals using US. However, to the best of the authors’ knowledge, no studies have been conducted on this topic.

On the basis of published research, clearly only a combination of US with heat or chemical sanitizers can sufficiently inactivate vegetative cells, spores, and enzymes simultaneously. Only then can consistent and high product quality be ensured, as the microbial enzymes can cause great damage to proteins and carbohydrates of the grains, even after killing of the vegetative cells [7].

## 6. High Hydrostatic Pressure (HHP)

Another emergent approach for the decontamination of food and feed products from spoilage microbes is treatment with high hydrostatic pressure (HHP). Well-established applications include the preservation of meat products, oysters, fruit juices, and many ready-to-eat foods. HHP may inactivate vegetative microorganisms and fungal spores at relatively low temperatures without compromising sensory and nutritional properties [92,93]. Thus, it has potential use in the expansion of the production of value-added foods.

Microbial inactivation is reported for processing pressures ranging from 100 to 800 MPa and relatively short times (a few seconds to several minutes). Combined treatment with mild temperatures between 20 and 50 °C to inactivate enzymes also have been reported. The treatment conditions depend fundamentally on the food matrix as well as the microorganisms and enzymes targeted. However, it is noteworthy that this technology cannot inactivate bacterial endospores with the application of the processing parameters commonly used in the food industry [94]. Pressures of at least 600 MPa with mild temperatures (60 °C) are required for the killing of spores of most food spoilage bacteria. Certain strains of *Clostridium botulinum* and *Bacillus species* are reported to withstand hydrostatic pressures of up to 1000 MPa [92]. Therefore, they present a much bigger challenge and cannot be inhibited by HHP alone, but which may be possible in combination with more severe heat treatment [95].

For the inactivation of *Escherichia coli* K-12 and *Staphylococcus aureus* ATCC 6538 in cheese slurries, 20 min of 400 MPa at 30 °C and of 600 MPa at 20 °C, respectively, were found to be sufficient [96]. Likewise, potential pathogenic food bacteria were inhibited due to HHP treatment. Studies on almonds, pressurised in water for 6 min at 414 MPa, followed by air drying at room temperature or 115 °C for 25 min, resulted in bacterial growth reduction of 4- and 6.7-log units, respectively [97]. The authors concluded that HHP treatments show great potential for microbial inactivation if the sample is suspended in the pressurizing medium.

With regards to fungal inhibition by HHP, Martínez-Rodríguez et al. [98] investigated the impact of HHP (300, 400, and 500 MPa, respectively) at 20 °C for 10 min on fungal mycelia development, spore viability, and enzymatic activity of *P. roqueforti*. Mycelia development was significantly reduced following all three treatments but in direct correlation with the applied pressure. Furthermore, the spore viability was notably reduced after exposure to 300 MPa and completely inhibited at higher pressures. Similarly, the total lipolytic activity of the samples decreased with increasing pressure. Researchers reported similar results for the inhibition of different food spoilage fungi, such as *Penicillium* spp., *Fusarium* spp., and *Aspergillus* spp., in a liquid medium and cheese [96,99]. This suggests that HHP treatments are a promising option for the decontamination of cereal grains in particular, as the grains are known to withstand pressures of 400 MPa without sensory or grain quality deterioration. Thus, potential spoilage fungi and their hydrolytic enzymes could be completely inhibited, without the need for classic heat treatments or chemicals. However, asco-spores of heat-resistant moulds were also reported to possess a high resistance to HHP. Hence, pressure treatments routinely applied to foods do not result in sufficient inhibition. However, HHP appears to sensitize asco-spores to subsequent heat treatments. Thus, a combination of heat and pressure treatment appears very promising [99].

Although the prevention of fungal growth is the best way to ensure mycotoxin-free crops, minor in-field contamination can result in mycotoxins present in otherwise good quality cereals. Thus, the in vitro and in situ degradation of mycotoxins is another relevant topic of research. However, few studies have investigated the sensitivity of mycotoxins to HHP treatments. The degradation of patulin in fruit juices was reported after treatment with 600 MPa for 300 s at 11 °C [100]. Unfortunately, there are no studies available regarding HHP treatment of cereals for microbial decontamination or degradation of mycotoxins. In addition, as observed previously by several researchers, the sensitivity of pathogenic and spoilage organisms and their metabolic products greatly depends on a number of factors, including the surrounding sample matrix, microbial strain, processing conditions, and moisture levels [92,95,96,99,101,102]. Therefore, without sufficient studies on cereal grains and investigations of typical cereal contaminants, it is impossible to predict the efficiency of HHP treatments on the microbial decontamination of cereal grains prior to storage.

In conclusion, the application of HHP appears to represent a promising approach for ensuring microbial safety without the need for chemical preservatives. However, HHP treatments require the sample to be suspended in a liquid medium. Otherwise, the pressure applied cannot be distributed evenly, leading to unsatisfactory results. This ultimately would require high moisture levels in the stored grains or would necessitate an additional drying step after the initial HHP treatment. HHP treatment has been shown to be effective in reducing the microbial load of foods for both pathogenic and spoilage microorganisms with minimal impact on the product quality. However, that the treatment is unsuitable for in-line procedures and must be performed in a batch process presents its biggest draw-back. Various parameters such as pressure, time, temperature, and pH have to be considered to optimise the process in terms of both microbial inactivation and in consideration of final product quality [103].

In particular, the combination of HHP with heating treatments requires further research to fully exploit its potential in the development of new products.

## 7. Conclusion and Future Trends

Scientific evidence for the potential of each treatment as a tool for microbial decontamination is available. In particular, novel technologies not currently used in industry were found to present several advantages over established ones. For example, the use of superheated steam combines a faster, more efficient microbial decontamination with less impact on the grain quality compared to conventional saturated steam or hot air treatments. In addition, the use of e-beam irradiation, high hydrostatic pressure, and microwaves, based on non-thermal inactivation mechanisms, presents several advantages over more established technologies, such as gamma rays or UV light. However, due to their novelty, particularly in terms of microbial decontamination of cereals, the side effects of these technologies have been sparsely investigated. Therefore, further research is required to better understand the impact on the treated grains and the effect on spoilage organisms after exposure to sub-lethal doses.

Another topic investigated in this review was the degradation of in-field produced and accumulated mycotoxins to prevent a potential consumer health hazard. All the evaluated treatments showed some potential for reducing the mycotoxin content in cereals. However, as a result of their structural diversity (Table 1) and higher intrinsic resistance against many treatments, when compared to the living organisms, the reduction of the myxotoxigenic load was found to be a major challenge. In many cases, the treatment time and intensity had to be much higher for mycotoxin degradation than for the inhibition of living organisms. Thus, successful degradation of these toxins is a major challenge without compromising the grain’s quality and seed viability. In addition, major concern related to the decay of mycotoxins are the degradation products released. The vast majority of research has examined the reduction in the level of the original parent mycotoxin, paying little attention to the degradation products or mechanism of degradation. Therefore, the identity of the degradation products and their toxicity is unknown. As a result, the efficiency of mycotoxin degradation cannot be evaluated as the released compounds may potentially be equal or even more toxic compared to the parent toxin. Future research will need to understand the possible degradation mechanisms to identify the resulting compounds. Only then will it be possible to assess their toxicity and evaluate the success and efficiency of the treatments discussed in this review. Furthermore, concerns regarding a possible reformation of the toxins during subsequent processing steps and the formation of masked mycotoxins due to the treatments merits greater research interest. 

For both purposes, microbial decontamination and detoxification, each treatment encompasses associated difficulties for the successful application to cereals. The even and homogeneous treatment of the whole sample appears to be the biggest challenge. In addition, the efficiency of a treatment depends on various influential factors. The sample matrix, target organism, moisture content, and water availability were the most frequently observed influencing factors within the available literature. Thus, the treatment conditions and setting must be optimised for each crop to make industrial application possible. However, even under ideal process conditions it appears unlikely that one physical treatment can result in sufficient microbial decontamination and detoxification without substantial grain quality deterioration. Consequently, the combination of several treatments appears to represent the most promising approach for optimal results. Future research should therefore focus on understanding and the optimisation of the synergies which are likely to be achievable through combinatory treatments. Only then will it be possible to produce products that meet the highest standards in terms of food quality and safety.

## Figures and Tables

**Table 1:** Chemical structures of mycotoxins commonly found on cereal grains sorted by the producing fungal species.

*Aspergillus* spp.	*Aspergillus* spp./*Penicillium* spp.	*Fusarium* spp.
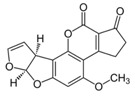		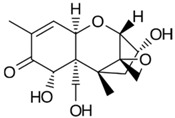
Aflatoxin B_1_		Deoxynivalenol
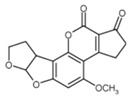	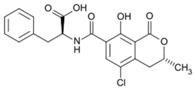	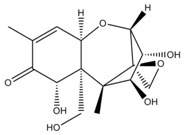
Aflatoxin B_2_	Ochratoxin A	Nivalenol
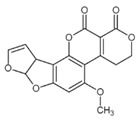	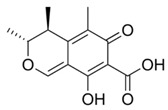	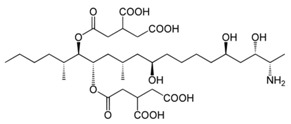
Aflatoxin G_1_	Citrinin	Fumonisin B_1_
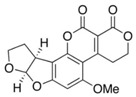		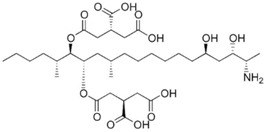
Aflatoxin G_2_		Fumonisin B_2_
		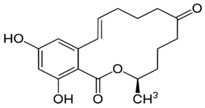
		Zearalenone

**Table 2:** The effects of different heat (wet and dry) treatments on microbial load and mycotoxin content in various sample matrices.

Target Organism/Toxin	Treatment	Sample Matrix	Technological Impact	References
Natural microbial load	Dry air 9 day/100 °C	Various cereals	No impact	[21,22]
Natural microbial load	Steam 60 min/82 °C	corn	No impact	[23]
Natural microbial load	Steam 210–250 °C/15 s	Wheat, barley, rye	Not investigated	[24]
*F. graminearum*	dry air 15 day/60 °C; 5 day/70 °C; 2 day/80 °C	Wheat	No impact	[25]
*F. graminearum*	Dry air 5 day/90 °C	Wheat	Reduced seed viability	[25]
*F. graminearum*	Dry air 21 day/60 °C; 9 day/70 °C; 5 day/80 °C	Barley	Reduced viability for 9 day/70 deg; 5 day/80 deg	[25]
*Aspergillus* spp., *Penicillium* spp., *Fusarium* spp., *E. coli*, *L. Monocytogenes*, *Salmonella* spp.	Steam 170–200 °C/<60 s	Various cereals	No impact	[26,27,28]
*Geobacillus stearothermophilus* spores	Steam 20 min/160 °C	Dried spore pellet-sand mixture	Not investigated	[29]
DON (50% reduction)	Steam 6 min/185 °C	wheat	Not investigated	[29]

**Table 3:** The effects of different ionizing irradiation treatments (gamma- and e-beam irradiation) on microbial load and mycotoxin content in various sample matrices.

Target Organism/Toxin	Treatment	Sample Matrix	Technological Impact	References
Natural fungal population	0.75 kGy gamma	millet	none	[44]
Natural microbial load	6 kGy e-beam	chestnuts	No effect on nutritional value	[45,46]
*L. monocytogenes*	3.3 kGy e-beam (soft electrons)	Alfalfa sprouts	No quality deterioration	[47]
*Aspergillus* spp., *Alternaria* spp., *Fusarium* spp., *Curvularia* spp., *Helminthosporium* spp.	1.5–3.5 kGy gamma	wheat	Reduced quality for doses >2.5 kGy	[48]
*Fusarium* spp.	4 kGy gamma	Barley	Reduced quality	[49]
*Fusarium* spp.	6 kGy gamma	Wheat and maize	Reduced quality	[49]
*Aspergillus* spp., *Penicillium* spp.	10–15 kGy e-beam	Dry split beans	No quality deterioration (10 kGy)	[50]
*Penicillium* spp., *Fusarium* spp., *Aspergillus* spp.	1.7–4.8 kGy e-beam	corn	Not investigated	[41]
*Fusarium* spp. and DON	6–10 kGy e-beam	Barley, malt	Not investigated	[51]
OTA and aflatoxins	15 kGy gamma	Wheat and sesame	Not investigated	[52,53,54]
OTA	2 kGy gamma	Aqueous solution	-	[55]
DON, ZEN, T-2, FB_1_	10 kGy gamma	Soy beans, corn, wheat	Not investigated	[56]
FB_1_	7 kGy gamma	Barley, wheat, maize	Not investigated	[49]
aflatoxins	1.5 kGy e-beam	Ground almond flour	Not investigated	[57]

DON: Desoxynivalenol; OTA: Ochratoxin A; ZEN: Zearalenone; T-2: Fusariotoxin T 2; FB_1_: Fumonisin B1; -: none.

**Table 4:** Comparison of gamma (^60^Co) and e-beam irradiation, adopted from Freita-Silva et al. [1].

Parameters	Gamma Irradiation	E-Beam Irradiation
Irradiation Time	Slow	Fast
Doses (kGy)	Higher doses	Lower doses
Source	Radioactive material	Electricity to generate electrons
Flexibility	Inflexible (cannot be turned off)	More flexible (can be turned off)
Penetration	Good penetration	Lower penetration power

**Table 5:** The effects of different non-ionizing (light, microwave, ultrasound) treatments on microbial load and mycotoxin content in various sample matrices.

Target Organism/Toxin	Treatment	Sample Matrix	Technological Impact	References
Different food spoilage bacteria and fungi	US (ultrasound) > 60 W/cm^2^	Aqueous solution	-	[73]
*A. parasiticus*	Microwave: 900 W, 2.45 GHz, 1–5 min	Aqueous solution	-	[74]
*Aspergillus* spp. and *Penicillium* spp.	US: 6 min, 60 °C, 20–39 W/cm^2^	Culture medium	-	[75]
*Aspergillus* spp.	51.2 J/g pulsed white light	wheat	15% reduced seed viability	[76]
*Aspergillus* spp.	Microwave: 120 s, 2450 MHz, 1.25 kW	Cereals and nuts	Not investigated	[77,78]
*Bacillus subtilis*	1.0 J/cm^2^ * 10 pulses light with 200–1100 nm	spices	No quality deterioration	[79]
OTA, OTB (Ochratoxin B), citrinin	Light: 455 nm/470 nm for 5 day	Aqueous solution	-	[80]
Aflatoxins	UV (Ultraviolet)-light: 265 nm for 15–45 min	nuts	Not investigated	[81]
trichothecenes	US > 200 W/cm^2^	corn	No quality deterioration	[82]

* Times (sign for multiplication), -: none.

## References

[1] Freita-Silva O., de Oliveira P.S., Freire Júnior M. (2014). Potential of Electron Beams to Control Mycotoxigenic Fungi in Food. Food Eng. Rev..

[2] Oerke E.-C. (2006). Crop losses to pests. J. Agric. Sci..

[3] Siciliano I., Spadaro D., Prelle A., Vallauri D., Cavallero M.C., Garibaldi A., Gullino M.L. (2016). Use of cold atmospheric plasma to detoxify hazelnuts from aflatoxins. Toxins.

[4] Magan N., Hope R., Cairns V., Aldred D. (2003). Post-harvest fungal ecology: Impact of fungal growth and mycotoxin accumulation in stored grain. Eur. J. Plant Pathol..

[5] Oliveira P.M., Zannini E., Arendt E.K. (2014). Cereal fungal infection, mycotoxins, and lactic acid bacteria mediated bioprotection: From crop farming to cereal products. Food Microbiol..

[6] Magan N., Aldred D. (2007). Post-Harvest Control Strategies: Minimizingmycotoxins in the Food Chain. Int. J. Food Microbiol..

[7] Schmidt M., Horstmann S., De Colli L., Danaher M., Speer K., Zannini E., Arendt E.K. (2016). Impact of fungal contamination of wheat on grain quality criteria. J. Cereal Sci..

[8] Audenaert K., Monbaliu S., Deschuyffeleer N., Maene P., Vekeman F., Haesaert G., De Saeger S., Eeckhout M. (2012). Neutralized electrolyzed water efficiently reduces *Fusarium* spp. in vitro and on wheat kernels but can trigger deoxynivalenol (DON) biosynthesis. Food Control.

[9] Cheli F., Pinotti L., Rossi L., Dell’Orto V. (2013). Effect of milling procedures on mycotoxin distribution in wheat fractions: A review. LWT—Food Sci. Technol..

[10] Vidal A., Marín S., Ramos A.J., Cano-Sancho G., Sanchis V. (2013). Determination of aflatoxins, deoxynivalenol, ochratoxin A and zearalenone in wheat and oat based bran supplements sold in the Spanish market. Food Chem. Toxicol..

[11] Magan N., Aldred D. (2006). Food Spoilage Microorganisms.

[12] Balasubramaniam (Bala) V.M., Martínez-Monteagudo S.I., Gupta R. (2015). Principles and Application of High Pressure–Based Technologies in the Food Industry. Annu. Rev. Food Sci. Technol..

[13] Crowley S., Mahony J., Van Sinderen D. (2013). Current perspectives on antifungal lactic acid bacteria as natural bio-preservatives. Trends Food Sci. Technol..

[14] Pawlowska A.M., Zannini E., Coffey A., Arendt E.K. (2012). “Green Preservatives”: Combating Fungi in the Food and Feed Industry by Applying Antifungal Lactic Acid Bacteria. Adv. Food Nutr. Res..

[15] Magan N., Lacey J. (1984). Effects of gas composition and water activity on growth of field and storage fungi and their interactions. Trans. Br. Mycol. Soc..

[16] Taniwaki M.H., Hocking A.D., Pitt J.I., Fleet G.H. (2001). Growth of fungi and mycotoxin production on cheese under modified atmospheres. Int. J. Food Microbiol..

[17] Gupta A., Sinha S.N., Atwal S.S. (2014). Modified Atmosphere Technology in Seed Health Management: Laboratory and Field Assay of Carbon Dioxide against Storage Fungi in Paddy. Plant Pathol. J..

[18] Savi G.D., Scussel V.M. (2014). Effects of Ozone Gas Exposure on Toxigenic Fungi Species from *Fusarium*, *Aspergillus*, and *Penicillium* Genera. Ozone Sci. Eng. J. Int. Ozone Assoc..

[19] O’Donnell C.P., Tiwari B.K., Bourke P., Cullen P.J. (2010). Effect of ultrasonic processing on food enzymes of industrial importance. Trends Food Sci. Technol..

[20] Klein J.D., Lurie S. (1991). Postharvest heat treatment and fruit quality. Postharvest News Inf..

[21] Lan S. (2006). Effects of Post-Harvest Treatment and Heat Stress on the Antioxidant Properties of Wheat. Master’s Thesis.

[22] Lehtinen P., Kiiliäinen K., Lehtomäki I., Laakso S. (2003). Effect of Heat Treatment on Lipid Stability in Processed Oats. J. Cereal Sci..

[23] Rose D.J., Bianchini A., Martinez B., Flores R.A. (2012). Methods for reducing microbial contamination of wheat flour and effects on functionality. Cereal Foods World.

[24] Bari L., Ohki H., Nagakura K., Ukai M. (2015). Application of Ultra Superheated Steam Technology (USST) to Food Grain Preservation at Ambient Temperature for Extended Periods of Time. Adv. Food Technol. Nutr. Sci..

[25] Gilbert J., Woods S.M., Turkington T.K., Tekauz A. (2005). Effect of heat treatment to control *Fusarium graminearum* in wheat seed. Can. J. Plant Pathol..

[26] Chang Y., Li X.-P., Liu L., Ma Z., Hu X., Zhao W., Gao G. (2015). Effect of Processing in Superheated Steam on Surface Microbes and Enzyme Activity of Naked Oats. J. Food Process. Preserv..

[27] Hu Y., Nie W., Hu X., Li Z. (2016). Microbial decontamination of wheat grain with superheated steam. Food Control.

[28] Ban G.-H., Kang D.-H. (2016). Effectiveness of superheated steam for inactivation of *Escherichia coli* O157:H7, *Salmonella* Typhimurium, *Salmonella* Enteritidis phage type 30, and *Listeria monocytogenes* on almonds and pistachios. Int. J. Food Microbiol..

[29] Cenkowski S., Pronyk C., Zmidzinska D., Muir W.E. (2007). Decontamination of food products with superheated steam. J. Food Eng..

[30] Clear R.M., Patrick S.K., Wallis R., Turkington T.K. (2002). Effect of dry heat treatment on seed-borne *Fusarium graminearum* and other cereal pathogens. Can. J. Plant Pathol..

[31] Bond W.W., Favero M.S., Petersen N.J., Marshall J.H. (1970). Dry-heat inactivation kinetics of naturally occurring spore populations. Appl. Microbiol..

[32] Nielsen K.F., Holm G., Uttrup L.P., Nielsen P.A. (2004). Mould growth on building materials under low water activities. Influence of humidity and temperature on fungal growth and secondary metabolism. Int. Biodeterior. Biodegrad..

[33] Miller J.D., Trenholm H.L. (1997). Mycotoxins in Grain Compounds Other than Aflatoxin.

[34] Bretz M., Knecht A., Göckler S., Humpf H.U. (2005). Structural elucidation and analysis of thermal degradation products of the *Fusarium* mycotoxin nivalenol. Mol. Nutr. Food Res..

[35] Vidal A., Sanchis V., Ramos A.J., Marín S. (2015). Thermal stability and kinetics of degradation of deoxynivalenol, deoxynivalenol conjugates and ochratoxin A during baking of wheat bakery products. Food Chem..

[36] Boudra H., Lebars P., Lebars J. (1995). Thermostability of Ochratoxin A in Wheat under 2 Moisture Conditions. Appl. Environ. Microbiol..

[37] Syamaladevi R.M., Tang J., Villa-Rojas R., Sablani S., Carter B., Campbell G. (2016). Influence of Water Activity on Thermal Resistance of Microorganisms in Low-Moisture Foods: A Review. Compr. Rev. Food Sci. Food Saf..

[38] Ban G.H., Yoon H., Kang D.H. (2014). A comparison of saturated steam and superheated steam for inactivation of *Escherichia coli* O157: H7, *Salmonella* Typhimurium, and *Listeria monocytogenes* biofilms on polyvinyl chloride and stainless steel. Food Control.

[39] Alfy A., Kiran B.V., Jeevitha G.C., Hebbar H.U. (2016). Recent Developments in Superheated Steam Processing of Foods—A Review. Crit. Rev. Food Sci. Nutr..

[40] Jalili M. (2015). A Review on Aflatoxins Reduction in Food. Iran. J. Health Saf. Environ..

[41] Nemţanu M.R., Braşoveanu M., Karaca G., Erper I. (2014). Inactivation effect of electron beam irradiation on fungal load of naturally contaminated maize seeds. J. Sci. Food Agric..

[42] Dev S.R.S., Birla S.L., Raghavan G.S.V., Subbiah J. (2012). Microbial decontamination of food by microwave (MW) and radiao frequency (RF). Microbial Decontamination in the Food Industry.

[43] (1982). FAO/IAEA Training Manual on Food Irradiation Technology and Techniques. Technical Reports Series, Proceedings of the International Atomic Energy Commission.

[44] Mahmoud N.S., Awad S.H., Madani R.M.A., Osman F.A., Elmamoun K., Hassan A.B. (2016). Effect of γ radiation processing on fungal growth and quality characteristcs of millet grains. Food Sci. Nutr..

[45] D’Ovidio K.L., Trucksess M.W., Devries J.W., Bean G. (2007). Effects of irradiation on fungi and fumonisin B1 in corn, and of microwave-popping on fumonisins in popcorn. Food Addit. Contam..

[46] Lung H.-M., Cheng Y.-C., Chang Y.-H., Huang H.-W., Yang B.B., Wang C.-Y. (2015). Microbial decontamination of food by electron beam irradiation. Trends Food Sci. Technol..

[47] Supriya P., Sridhar K.R., Ganesh S. (2014). Fungal decontamination and enhancement of shelf life of edible split beans of wild legume Canavalia maritima by the electron beam irradiation. Radiat. Phys. Chem..

[48] Salem E.A., Soliman S.A., El-Karamany A.M., El-shafea Y.M.A. (2016). Effect of Utilization of Gamma Radiation Treatment and Storage on Total Fungal Count, Chemical Composition and Technological Properties Wheat Grain. Egypt. J. Biol. Pest Control.

[49] Aziz N.H., El-Far F.M., Shahin A.A.M., Roushy S.M. (2007). Control of *Fusarium* moulds and fumonisin B1 in seeds by gamma-irradiation. Food Control.

[50] Carocho M., Antonio A.L., Barreira J.C.M., Rafalski A., Bento A., Ferreira I.C.F.R. (2014). Validation of Gamma and Electron Beam Irradiation as Alternative Conservation Technology for European Chestnuts. Food Bioprocess Technol..

[51] Carocho M., Barros L., Antonio A.L., Barreira J.C.M., Bento A., Kaluska I., Ferreira I.C.F.R. (2013). Analysis of organic acids in electron beam irradiated chestnuts (*Castanea sativa* Mill.): Effects of radiation dose and storage time. Food Chem. Toxicol..

[52] Akueche E.C., Anjorin S.T., Harcourt B.I., Kana D. (2012). Studies on fungal load, total aflatoxins and ochratoxin a contents of gamma-irradiated and non-irradiated Sesamum indicum grains from Abuja markets, Nigeria. Kasetsart J. Nat. Sci..

[53] Herzallah S., Alshawabkeh K., Al Fataftah A. (2008). Aflatoxin decontamination of artificially contaminated feeds by sunlight, γ-radiation, and microwave heating. J. Appl. Poult. Res..

[54] Jalili M., Jinap S., Noranizan M.A. (2012). Aflatoxins and ochratoxin a reduction in black and white pepper by gamma radiation. Radiat. Phys. Chem..

[55] Mehrez A., Maatouk I., Romero-González R., Ben Amara A., Kraiem M., Garrido Frenich A., Landoulsi A. (2016). Assessment of ochratoxin A stability following gamma irradiation: Experimental approaches for feed detoxification perspectives. World Mycotoxin J..

[56] Hooshmand H., Klopfenstein C.F. (1995). Effects of gamma irradiation on mycotoxin disappearance and amino acid contents of corn, wheat, and soybeans with different moisture contents. Plant Foods Hum. Nutr..

[57] Carocho M., Barreira J.C., Antonio A.L., Bento A., Kaluska I., Ferreira I.C. (2012). Effects of electron beam radiation on nutritional parameters of Portuguese chestnuts (*Castanea sativa* mill.). J. Agric. Food Chem..

[58] El-Naggar S.M., Mikhaiel A.A. (2011). Disinfestation of stored wheat grain and flour using gamma rays and microwave heating. J. Stored Prod. Res..

[59] Melki M., Marouani A. (2010). Effects of gamma rays irradiation on seed germination and growth of hard wheat. Environ. Chem. Lett..

[60] Deberghes P., Betbeder A.M., Boisard F., Blanc R., Delaby J.F., Krivobok S., Steiman R., Seigle-Murandi F., Creppy E.E. (1995). Detoxification of ochratoxin A, a food contaminant: Prevention of growth of *Aspergillus ochraceus* and its production of ochratoxin A. Mycotoxin Res..

[61] Di Stefano V., Pitonzo R., Cicero N., D’Oca M.C. (2014). Mycotoxin contamination of animal feedingstuff: Detoxification by gamma-irradiation and reduction of aflatoxins and ochratoxin A concentrations. Food Addit. Contam. Part A.

[62] Aquino S., Ferreira F., Ribeiro D.H.B., Corrêa B., Greiner R., Villavicencio A.L.C.H. (2005). Evaluation of viability of *Aspergillus flavus* and aflatoxins degradation in irradiated samples of maize. Braz. J. Microbiol..

[63] Farag R.S., El-Baroty G.S., Abo-Hagger A.A. (2004). Aflatoxin destruction and residual toxicity of contaminated-irradiated yellow corn and peanuts on rats. Adv. Food Sci..

[64] Prado G., de Carvalho E.P., Oliveira M.S., Madeira J.G.C., Morais V.D., Correa R.F., Cardoso V.N., Soares T.V., da Silva J.F.M., Gonçalves R.C.P. (2003). Effect of gamma irradiation on the inactivation of aflatoxin B1 and fungal flora in peanut. Braz. J. Microbiol..

[65] Wang F., Xie F., Xue X., Wang Z., Fan B., Ha Y. (2011). Structure elucidation and toxicity analyses of the radiolytic products of aflatoxin B_1_ in methanol-water solution. J. Hazard. Mater..

[66] Kume T., Furuta M., Todoriki S., Uenoyama N., Kobayashi Y. (2009). Status of food irradiation in the world. Radiat. Phys. Chem..

[67] Kottapalli B., Wolf-Hall C.E., Schwarz P. (2006). Effect of electron-beam irradiation on the safety and quality of *Fusarium*-infected malting barley. Int. J. Food Microbiol..

[68] Stepanik T., Kost D., Nowicki T., Gaba D. (2007). Effects of electron beam irradiation on deoxynivalenol levels in distillers dried grain and solubles and in production intermediates. Food Addit. Contam..

[69] Lanza C.M., Mazzaglia A., Paladino R., Auditore L., Barnà D., Loria D., Trifirò A., Trimarchi M., Bellia G. (2013). Characterization of peeled and unpeeled almond (*Prunus amygdalus*) flour after electron beam processing. Radiat. Phys. Chem..

[70] Schoeller N.P., Ingham S.C., Ingham B.H. (2002). Assessment of the Potential for *Listeria monocytogenes* Survival and Growth during Alfalfa Sprout Production and Use of Ionizing Radiation as a Potential Intervention Treatment. J. Food Prot..

[71] Kikuchi O.K., Todoriki S., Saito M., Hayashi T. (2003). Efficacy of soft-electron (low-energy electron beam) for soybean decontamination in comparison with gamma-rays. J. Food Sci..

[72] Farkas J., Mohacsi-Farkas C. (2011). History and future of food irradiation. Trends Food Sci. Technol..

[73] Oms-Oliu G., Martín-Belloso O., Soliva-Fortuny R. (2010). Pulsed light treatments for food preservation. A review. Food Bioprocess Technol..

[74] Aron Maftei N., Ramos-Villarroel A.Y., Nicolau A.I., Martín-Belloso O., Soliva-Fortuny R. (2014). Pulsed light inactivation of naturally occurring moulds on wheat grain. J. Sci. Food Agric..

[75] Nicorescu I., Nguyen B., Moreau-Ferret M., Agoulon A., Chevalier S., Orange N. (2013). Pulsed light inactivation of Bacillus subtilis vegetative cells in suspensions and spices. Food Control.

[76] Schmidt-Heydt M., Cramer B., Graf I., Lerch S., Hunpf H.-U., Geisen R. (2012). Wavelength-dependent degradation of ochratoxin and citrinin by light in vitro and in vivo and its implications on *Penicillium*. Toxins.

[77] Jubeen F., Bhatti I.A., Khan M.Z., Shahid M. (2012). Effect of UVC Irradiation on Aflatoxins in Ground Nut (*Arachis hypogea*) and Tree Nuts (*Juglans regia*, *Prunus duclus* and *Pistachio vera*). Chem. Soc. Pak..

[78] Liu R., Jin Q., Tao G., Shan L., Huang J., Liu Y., Wang X., Mao W., Wang S. (2010). Photodegradation kinetics and byproducts identification of the Aflatoxin B1 in aqueous medium by ultra-performance liquid chromatography-quadrupole time-of-flight mass spectrometry. J. Mass Spectrom..

[79] Liu R., Chang M., Jin Q., Huang J., Liu Y., Wang X. (2011). Degradation of aflatoxin B1 in aqueous medium through UV irradiation. Eur. Food Res. Technol..

[80] Fang Y., Hu J., Xiong S., Zhao S. (2011). Effect of low-dose microwave radiation on *Aspergillus parasiticus*. Food Control.

[81] Ursu M.-P. (2015). Usage of Microwaves for Decontamination of Sensible Materials and Cereal Seeds. Rev. Tehnol. Neconv..

[82] Kabak B., Dobson A.D., Var I. (2006). Strategies to Prevent Mycotoxin Contamination of Food and Animal Feed: A Review. Crit. Rev. Food Sci. Nutr..

[83] Basaran P., Akhan Ü. (2010). Microwave irradiation of hazelnuts for the control of aflatoxin producing *Aspergillus parasiticus*. Innov. Food Sci. Emerg. Technol..

[84] Feng H., Yang W., Hielscher T. (2008). Power Ultrasound. Food Sci. Technol. Int..

[85] Butz P., Tauscher B. (2002). Emerging technologies: Chemical aspects. Food Res. Int..

[86] Chemat F., Zill-E-Huma, Khan M.K. (2011). Applications of ultrasound in food technology: Processing, preservation and extraction. Ultrason. Sonochem..

[87] Bilek S.E., Turantaş F. (2013). Decontamination efficiency of high power ultrasound in the fruit and vegetable industry, a review. Int. J. Food Microbiol..

[88] Scouten A.J., Beuchat L.R. (2002). Combined effects of chemical, heat and ultrasound treatments to kill *Salmonella* and *Escherichia coli* O157:H7 on alfalfa seeds. J. Appl. Microbiol..

[89] Seymour I.J., Burfoot D., Smith R.L., Cox L.A., Lockwood A. (2002). Ultrasound decontamination of minimally processed fruits and vegetables. Int. J. Food Sci. Technol..

[90] Herceg Z., Jambrak R.R., Vukušić T., Stulić V., Stanzer D., Milošević S. (2015). The effect of high-power ultrasound and gas phase plasma treatment on *Aspergillus* spp. and *Penicillium* spp. count in pure culture. J. Appl. Microbiol..

[91] Lindner W., Hasenhuti K. Decontamination and Detoxification of Corn Which Was Contaminated with Trichothecenes Applying Ultrasonication (Abstr.). Proceedings of the IX Internat IUPAC Symposium on Mycotoxins and Phytotoxins.

[92] Heinz V., Buckow R. (2009). Food preservation by high pressure. J. Verbrauch. Lebensmittelsich..

[93] Polydera A.C., Stoforos N.G., Taoukis P.S. (2003). Comparative shelf life study and vitamin C loss kinetics in pasteurised and high pressure processed reconstituted orange juice. J. Food Eng..

[94] Wannasawat Ratphitagsanti M. (2009). Approaches for Enhancing Lethality of Bacterial Spores Treated by Pressure-Assisted Thermal Processing.

[95] Patterson M.F. (2005). Microbiology of pressure-treated foods. J. Appl. Microbiol..

[96] O’Reilly C.E., O’Connor P.M., Kelly A.L., Beresford T.P., Murphy P.M. (2000). Use of hydrostatic pressure for inactivation of microbial contaminants in cheese. Appl. Environ. Microbiol..

[97] Willford J., Mendonca A., Goodridge L. (2008). Water Pressure Effectively Reduces Salmonella enterica Serovar Enteritidis on the Surface of Raw Almonds. J. Food Prot..

[98] Bello E.F.T., Martínez G.G., Klotz Ceberio B.F., Rodrigo D., López A.M. (2014). High Pressure Treatment in Foods. Foods.

[99] Black E.P., Setlow P., Hocking A.D., Stewart C.M., Kelly A.L., Hoover D.G. (2007). Response of spores to high-pressure processing. Compr. Rev. Food Sci. Food Saf..

[100] Hao H., Zhou T., Koutchma T., Wu F., Warriner K. (2016). High hydrostatic pressure assisted degradation of patulin in fruit and vegetable juice blends. Food Control.

[101] Martínez-Rodríguez Y., Acosta-Muñiz C., Olivas G.I., Guerrero-Beltrán J., Rodrigo-Aliaga D., Mujica-Paz H., Welti-Chanes J., Sepulveda D.R. (2014). Effect of high hydrostatic pressure on mycelial development, spore viability and enzyme activity of *Penicillium Roqueforti*. Int. J. Food Microbiol..

[102] Smith K., Mendonca A., Jung S. (2009). Impact of high-pressure processing on microbial shelf-life and protein stability of refrigerated soymilk. Food Microbiol..

[103] Torres J.A., Saraiva J.A., Guerra-Rodríguez E., Aubourg S.P., Vázquez M. (2014). Effect of combining high-pressure processing and frozen storage on the functional and sensory properties of horse mackerel (*Trachurus trachurus*). Innov. Food Sci. Emerg. Technol..

